# Incidence and Predictors of Opportunistic Infection Among People Living With HIV in Ethiopia: A Systematic Review and Meta‐Analysis

**DOI:** 10.1155/arat/7249878

**Published:** 2026-01-31

**Authors:** Beyene Zewdu Nigatu, Amhasilasie Ewunetu Ageze

**Affiliations:** ^1^ Clinical Governance and Quality Improvement Unit, Kobo Primary Hospital, Kobo, Ethiopia; ^2^ Department of Surgery, Kobo Primary Hospital, Kobo, Ethiopia

**Keywords:** Ethiopia, incidence, meta-analysis, opportunistic infections, predictors, systematic review

## Abstract

**Background:**

Opportunistic infections (OIs) play a crucial role in the morbidity and mortality of HIV‐infected individuals. Despite the increasing use of antiretroviral therapy, there remains a lack of comprehensive data on the incidence of OIs to provide a clearer national picture. Therefore, this systematic review and meta‐analysis aim to determine the pooled incidence and identify the predictors of OIs among people living with HIV in Ethiopia.

**Method:**

A systematic search was conducted across multiple electronic databases to identify relevant studies. The degree of heterogeneity across studies was assessed using the *I* ^2^ statistic. Subgroup analyses were conducted to explore sources of heterogeneity. A funnel plot and Egger’s test were used to assess publication bias. Adjusted hazard ratio with 95% CI was used to assess the relationship between predictors and OIs occurrence.

**Result:**

In total, 24 studies met the inclusion criteria and were analyzed. The pooled incidence rate of OIs among people living with HIV in Ethiopia was found to be 6.96 per 100 person‐years (95% CI: 5.14–8.78) based on a random‐effects model. The meta‐analysis identified predictors of OIs among people living with HIV, including poor adherence (pooled AHR 1.42, 95% CI: 1.12, 1.80), CD4 count < 200 cells/mm^3^ (AHR 1.49, 95% CI: 1.25, 1.78), being bedridden (AHR 1.45, 95% CI: 1.10, 1.90), and advanced WHO clinical stage (AHR 1.57, 95% CI: 1.24, 1.98).

**Conclusion:**

The burden of OIs continues to be a major health concern among people living with HIV/AIDS. Low CD4 count, poor adherence to ART, being bedridden, and advanced clinical stage were significantly associated with the occurrence of OIs.

## 1. Background

According to data from joint United Nations Programme on HIV/AIDS (UNAIDS), in 2023, an estimated 39.9 million people were living with HIV globally. There were approximately 1.3 million new infections and 630,000 HIV‐related deaths during the year [1]. Despite notable progress in HIV treatment through the broad rollout of antiretroviral therapy (ART), opportunistic infections (OIs) continue to be a major cause of illness and death among people living with HIV (PLWH), especially in low‐ and middle‐income countries like Ethiopia [2–5]. In Ethiopia, the incidence of OIS among HIV patients ranges between 5 and 15 cases per 100 person‐years (PYs) [2, 5–8].

PLWH are at risk of developing OIs; however, the prevalence and incidence of HIV‐associated OIs vary widely [9]. OIs are infections that occur more frequently and are more severe in people with weakened immune systems, including people with HIV [10, 11]. OIs frequently indicate the progression of HIV to acquired immunodeficiency syndrome (AIDS). Conditions such as tuberculosis (TB), pneumocystis pneumonia (PCP), cryptococcal meningitis, and cytomegalovirus retinitis are closely linked to severe immune system compromise [12]. The most common OIs in PLWH include TB, oral candidiasis, PCP, herpes zoster, toxoplasmosis, cytomegalovirus infection, and *Mycobacterium avium* complex [13].

All individuals infected with HIV are at risk of developing a variety of OIs [3]. OIs play a crucial role in the morbidity and mortality of HIV‐infected individuals, responsible for 94.1% of deaths related to HIV [14, 15].

Several factors have been strongly linked to the occurrence of OIs among individuals living with HIV, including poor adherence to ART, advanced WHO clinical staging, and severe immunosuppression [2, 16, 17].

The World Health Organization (WHO) has provided several key recommendations to reduce the mortality, hospitalizations, higher healthcare costs, and prolonged inability to work associated with OIs among PLWH. These strategies focus on prevention, early diagnosis, treatment, and strengthening healthcare systems [3, 12]. Ethiopian Ministry of Health (MOH) has developed several strategies and recommendations to reduce the impact of OIs among HIV‐infected individuals. These strategies include early HIV diagnosis and ART initiation, prophylaxis for common OIs, and integration of HIV care services [18, 19].

While numerous studies have examined the incidence and predictors of OIs among PLWH in Ethiopia, the findings are often fragmented, inconsistent, and region‐specific. These studies frequently vary in methodology, sample size, and population characteristics, which limit the ability to generalize results across the country. Despite the increasing use of ART, there remains a lack of comprehensive data that combine results from multiple regions and diverse patient groups to provide a clearer national picture. Despite the availability of several systematic reviews and meta‐analyses, important limitations remain in the existing evidence. Previous systematic reviews and meta‐analyses have focused on specific age groups that are either on children or adults, resulting in fragmented findings that cannot be generalized across the broader population. In addition, previous systematic reviews and meta‐analyses have concentrated on estimating the prevalence of OIs rather than the incidence of OIs. Therefore, a comprehensive review that synthesizes data across all age groups and directly assesses the incidence of OIs is still lacking. This gap in evidence hinders the development of effective, targeted strategies for preventing and managing OIs in PLWH. Therefore, this systematic review and meta‐analysis aim to determine the pooled incidence and identify the predictors of OIs among PLWH in Ethiopia.

## 2. Methods

### 2.1. Search Strategy

The search strategy for this systematic review and meta‐analysis follows a structured approach to ensure comprehensive coverage of the relevant literature. The strategy was conducted in alignment with the Preferred Reporting Items for Systematic Reviews and Meta‐Analyses (PRISMA) guidelines. This systematic review and meta‐analysis protocol has been registered with the International Prospective Register of Systematic Reviews (PROSPERO) under the registration number CRD42023489216.

A systematic search was conducted across multiple electronic databases to identify relevant studies. The databases to be searched include the following: PubMed, Google Scholar, and Directory of Open Access Journals. To identify relevant studies, the following search terms were utilized in the PubMed database: (“HIV”[MeSH Terms] OR “HIV Infections”[MeSH Terms] OR HIV[Title/Abstract] OR “people living with HIV”[Title/Abstract]) AND (“Opportunistic Infections”[MeSH Terms] OR “opportunistic infection∗”[Title/Abstract]) AND (“Incidence”[MeSH Terms] OR incidence[Title/Abstract] OR “predictors”[Title/Abstract] OR “risk factors”[Title/Abstract]) AND (Ethiopia[MeSH Terms] OR Ethiopia[Title/Abstract].

### 2.2. Eligibility Criteria

Two independent reviewers (BZ and AE) were first screened the titles and abstracts of all identified articles. The screening was done based on the inclusion and exclusion criteria. The full texts of all potentially eligible articles were retrieved and reviewed by the same two reviewers. Any disagreements between reviewers were resolved through discussion.

### 2.3. Inclusion Criteria

Studies were included in the review if they met the following criteria:

➢ Studies involving PLWH of any age group (adults, adolescents, or children) residing in Ethiopia.
➢ Studies that report on the incidence and/or predictors (risk factors or determinants) of one or more OIs (e.g., TB, oral candidiasis, PCP, and cryptococcal meningitis).
➢ Observational studies including cohort, cross‐sectional, or case–control designs, as well as clinical trials.
➢ Facility‐based or community‐based studies conducted in Ethiopia.
➢ Language: Articles published in English.

### 2.4. Exclusion Criteria

Studies were excluded based on the following:

➢ Studies not involving HIV‐positive individuals or studies conducted outside of Ethiopia
➢ Studies that do not report on either incidence or predictors of OIs
➢ Studies lacking adequate data for extraction or analysis, even after attempts to contact the authors
➢ Duplicate publications of the same study (the most complete or latest version will be retained)

### 2.5. Outcome Measure

The primary outcome of this review is the pooled incidence rate of OIs among PLWH in Ethiopia. Incidence was measured as the number of new OI cases per 100 PYs of follow‐up, as reported in each included study. When not directly provided, incidence rates were calculated using the number of OI events and the total person‐time at risk. The secondary outcome is to identify and pool predictors or risk factors associated with the development of OIs.

### 2.6. Quality Assessment

The methodological quality of the included studies was evaluated using standardized tools tailored to retrospective cohort studies; the Newcastle–Ottawa Scale (NOS) was applied. Two reviewers (BZ and AE) independently performed the quality assessments, and any disagreements were resolved through discussion. The results were used to evaluate the robustness of the findings and guide interpretation.

### 2.7. Certainty of Evidence

The certainty of evidence for each primary outcome was evaluated using the GRADE (Grading of Recommendations, Assessment, Development, and Evaluation) approach. This framework considers five key domains: risk of bias, inconsistency, indirectness, imprecision, and publication bias. Each outcome was assessed and rated as having high, moderate, low, or very low certainty of evidence [20]. Two reviewers (BZ and AE) independently conducted the GRADE assessment, with disagreements resolved through discussion.

### 2.8. Data Extraction Process

Microsoft Excel was prepared for data extraction as a template. Two reviewers (BZ and AE) independently extracted data from each included study. For each study, data were extracted on the authors, year of publication, study design, sample size, incidence and predictors of opportunistic infections (OIs), and duration of follow‐up. Any discrepancies were identified between the reviewers during data extraction was resolved through discussion.

### 2.9. Data Analysis

Data for this meta‐analysis were extracted from eligible studies based on the inclusion and exclusion criteria. Each study was carefully reviewed to ensure that the necessary information was collected. Data extraction was carried out using a predesigned Microsoft Excel template, and the extracted data were subsequently imported into Stata Version 17 for the meta‐analysis.

The data analysis was performed using a random‐effects model due to the expected heterogeneity across studies. For each study, the incidence rate of OIs was calculated as PYs incidence rate. The random‐effects model was used to pool the incidence rates from the individual studies. The pooled incidence rate was calculated along with a 95% confidence interval (CI).

Subgroup analyses were conducted to explore sources of heterogeneity based on region and duration of follow‐up. The degree of heterogeneity across studies was assessed using the *I* ^2^ statistic. Sensitivity analyses were conducted to assess the robustness of the findings. A funnel plot was created to visually inspect for potential publication bias, which can occur if studies with positive results are more likely to be published. Egger’s test was also used to test for asymmetry in the funnel plot, which would indicate bias. Adjusted hazard ratio (AHR) with 95% CI was extracted, to assess the relationship between predictors and OI occurrence.

The pooled incidence rate of OIs among PLWH was presented with a 95% CI. The pooled AHR with *p* values for each predictor was reported.

## 3. Results

### 3.1. Study Selection

**FIGURE 1:**
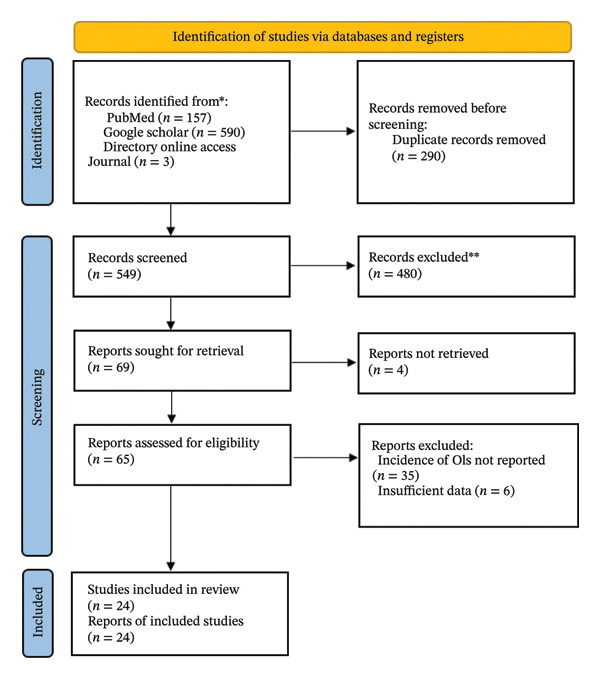
PRISMA 2020 flow diagram illustrating the selection process of studies.

A total of 750 studies were initially identified through database searches conducted in PubMed, Google Scholar, and Directory of Open Access Journals, along with additional records retrieved from reference lists and gray literature. After removing 201 duplicate records, 549 articles remained for title and abstract screening. Of these, 480 studies were excluded for not meeting the eligibility criteria. The full texts of 69 articles were then assessed in detail, resulting in the inclusion of 24 studies in the final systematic review and meta‐analysis (Figure 1).

### 3.2. Characteristics of Included Studies

The 24 studies included in this meta‐analysis were conducted between 2015 and 2024, involving a total of 11,167 PLWH across Ethiopia. Sample sizes varied from 271 to 816 participants, and the studies were conducted in diverse regions, including Addis Ababa, Amhara, Oromia, and South Ethiopia. All of the studies employed retrospective cohort designs and focused on assessing the incidence of OIs as well as identifying associated risk factors among PLWH. A detailed overview of the study characteristics, including author, year, region, study design, and sample size, is presented (Table 1).

**TABLE 1:** Summary of included study characteristics.

Author	Publication year	Region	Study design	Sample size	Case	Incidence
Tegegne et al. [21]	2022	Amhara	Retrospective cohort	354	114	13.5/100 PY
Woldegeorgis et al. [8]	2022	South Ethiopia	Retrospective cohort	515	164	8.97/100 PY
Dagnaw et al. [6]	2023	Amhara	Retrospective cohort	715	545	4.1/10,000 PY
Girma et al. [16]	2022	Oromia	Retrospective cohort	419	199	23/1000 PY
Melkamu et al. [5]	2020	Amhara	Retrospective cohort	408	129	9.7/100 PY
Mekonnen et al. [22]	2023	Amhara	Retrospective cohort	452	120	8.64/100 PY
Admasu et al. [23]	2024	Southwest Ethiopia	Retrospective cohort	409	122	12.8/100 PY
Kerebeh et al. [24]	2024	Amhara	Retrospective cohort	403	112	7.06/100 PY
Ayalaw et al. [25]	2015	Amhara	Retrospective cohort	271	52	4.9/100 PY
Alemu et al. [26]	2016	North Ethiopia	Retrospective cohort	645	79	4.2/100 PY
Jerene et al. [27]	2019	Addis Ababa and South Ethiopia	Retrospective cohort	816	64	2.25/100 PY
Endalamaw et al. [28]	2018	Amhara	Retrospective cohort	352	34	2.63/100 PY
Beshir et al. [29]	2019	Oromia	Retrospective cohort	428	67	6.03/100 PY
Mequanente et al. [30]	2022	Amhara	Retrospective cohort	389	219	4.2/100 PY
Kebede et al. [31]	2022	Benishangul‐Gumuze	Retrospective cohort	721	63	5.86/100 PY
Wondifraw et al. [32]	2022	Amhara	Retrospective cohort	341	129	6/100 PY
Mekonnen et al. [33]	2024	Amhara	Retrospective cohort	407	56	4.55/100 PY
Tekese et al. [34]	2022	South Ethiopia	Retrospective cohort	389	59	3.5/100 PY
Chanie et al. [35]	2020	Amhara	Retrospective cohort	349	87	5.53/100 PY
Ajema et al. [36]	2024	South Ethiopia	Retrospective cohort	393	54	6.26/100 PY
Bekele et al. [37]	2017	South Ethiopia	Retrospective cohort	554	160	8.79/100 PY
Ahmed et al. [38]	2018	Afar	Retrospective cohort	451	118	8.6/100 PY
Temesgen et al. [39]	2019	Amhara	Retrospective cohort	492	83	6.5/10 PY
Aemro et al. [40]	2020	Amhara	Retrospective cohort	494	62	6.19/100 PY

### 3.3. Quality of Included Study

A total of 24 retrospective cohort studies were included in this meta‐analysis. According to the NOS, the included studies were of moderate to high quality. In general, the studies provided clear definitions of exposures and outcomes. The NOS scores ranged from 7 to 9, with a mean score of 8.

### 3.4. Incidence of OIs

**FIGURE 2:**
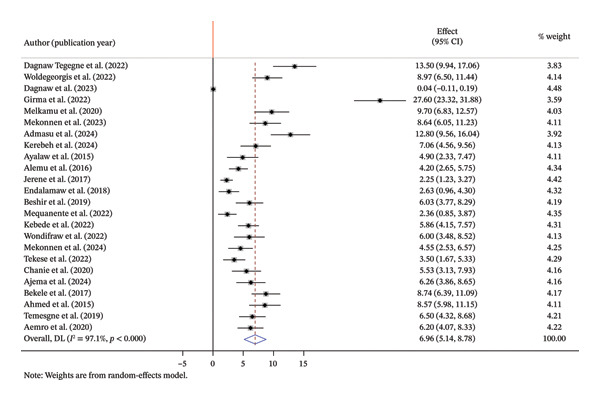
Forest plot summary of the pooled incidence of opportunistic infections among HIV patients in Ethiopia.

The pooled incidence rate of OIs among PLWH in Ethiopia was found to be 6.96 per 100 PYs (95% CI: 5.14–8.78) based on a random‐effects model. Incidence rates reported across the individual studies showed considerable variation, ranging from 0.04 to 27.6 per 100 PYs. Significant heterogeneity was observed across the included studies (*I* ^2^ = 97.1%, *p* < 0.001) (Figure 2).

### 3.5. Subgroup Analysis

**FIGURE 3:**
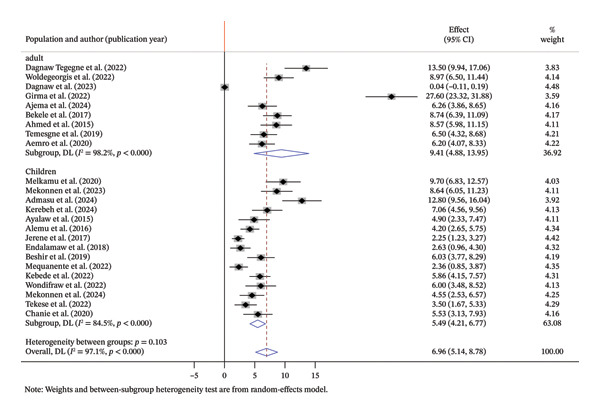
Summary of subgroup analysis based on population.

**FIGURE 4:**
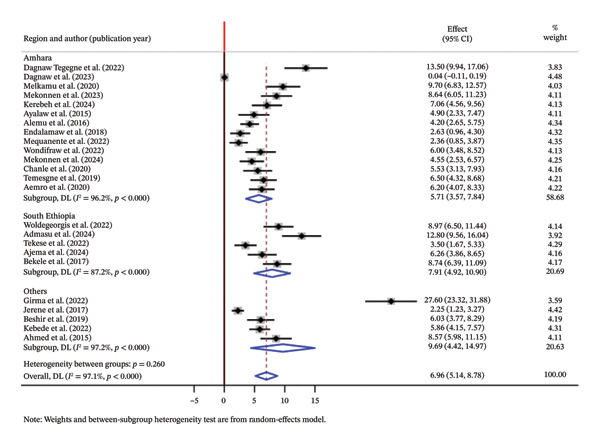
Summary of subgroup analysis based on region.

To explore potential sources of heterogeneity, subgroup analyses were performed based on the following factors:

➢ Population: The incidence of OIs among adult HIV patients was 9.41/100 PYs, and the incidence of OIs among children with HIV was 5.49/100 PYs (Figure 3).
➢ Study region: Incidence in Amhara was 5.71/100 PYs, while in South Ethiopia and others like Oromia, Benishangul‐Gumuze, and Afar, it was 7.91/100 PYs and 9.69/100 PYs, respectively (Figure 4).

### 3.6. Meta‐Regression

In the univariable meta‐regression, none of the examined study‐level characteristics including sample size (*p* = 0.233) and length of follow‐up (*p* = −0.58) were significantly associated with the effect size. These results suggest that heterogeneity in effect estimates could not be explained by the variables included (Table 2).

**TABLE 2:** Summary of meta‐regression analysis based on different covariates.

Incidence	Coefficient	Std. err.	*t*	*p* > *t*	[95% conf. interval]	
Sample size	−0.0103194	0.0079525	−1.30	0.208	−0.0268575	0.0062187
Length of follow‐up	−0.2284747	0.3050044	−0.75	0.462	−0.862766	0.4058167
_c _cons	13.5408	4.72948	2.86	0.009	3.705308	23.37629

### 3.7. Publication Bias

**FIGURE 5:**
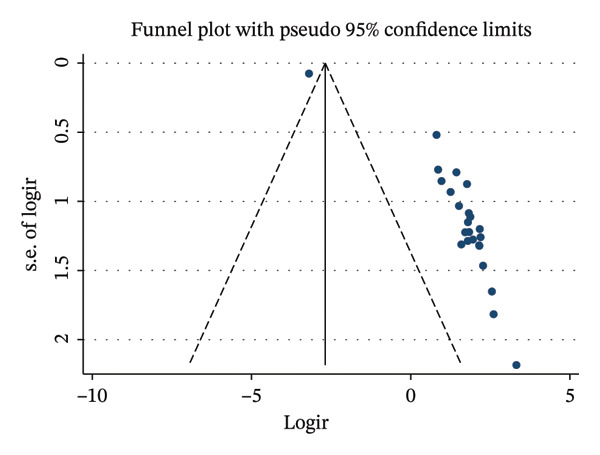
Funnel plot illustrating potential publication bias among the included studies.

To assess the potential for publication bias, we visually inspected the funnel plot for asymmetry. The plot, which is typically symmetric in the absence of publication bias, showed asymmetry in this analysis (Figure 5). The asymmetry suggests a possibility of publication bias, indicating that smaller studies with negative or null findings may have been underreported.

### 3.8. Statistical Tests for Publication Bias

To further investigate the presence of publication bias, we performed Egger’s test and Begg’s test. The result of Egger’s regression test was a *p* value of 0.001, which suggests the presence of significant publication bias. Begg’s rank correlation test returned a *p* value of 0.001, indicating the presence of small‐study effects.

### 3.9. Trim‐and‐Fill Analysis

Trim‐and‐fill analysis was performed to assess the potential impact of publication bias. The adjusted pooled effect size remained unchanged after the procedure (6.999, 95% CI: 4.991–9.006), indicating no evidence of missing studies due to publication bias and supporting the stability of the pooled estimate (Table 3).

**TABLE 3:** Summary of trim and fill analysis.

Studies	Effect size	[95% conf. interval]	
Observed	6.999	4.991	9.006
Observed + imputed	6.999	4.991	9.006

### 3.10. Predictors of OI

**FIGURE 6:**
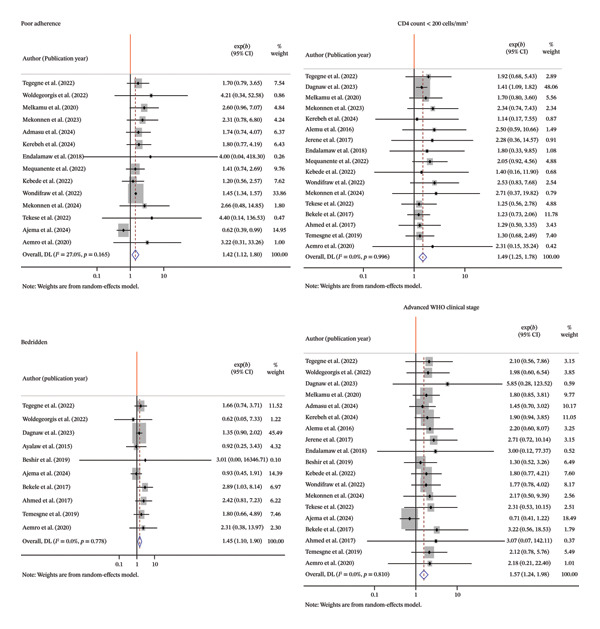
Forest plot illustrating the association different predictors and the occurrence of OIs based on a random‐effect model.

The meta‐analysis identified several key predictors of OIs among PLWH: poor adherence (pooled AHR 1.42, 95% CI: 1.12, 1.80), CD4 count < 200 cells/mm^3^ (AHR 1.49, 95% CI: 1.25, 1.78), being bedridden (AHR 1.45, 95% CI: 1.10, 1.90), and advanced WHO clinical stage (Stages 3 and 4) (AHR 1.57, 95% CI: 1.24, 1.98). Heterogeneity among studies ranged from low to minimal (*I* ^2^: 0%–27%) (Figure 6). Random‐effects models were used for all pooled estimates.

## 4. Discussion

In this meta‐analysis, we assessed the incidence and predictors of OI among PLWH in Ethiopia. Our findings indicate that the pooled incidence of OIs among HIV patients was 6.96/100 PYs (95% CI: 5.14, 8.78). This finding is consistent with those of several primary studies included in the analysis, which reported an incidence rate ranging from 5.53 to 8.7/100 PYs [22, 29, 31, 32, 35–41]. However, some studies showed higher incidence rates, ranging from 8.97 to 27/100 PYs [8, 16, 21, 23]. The possible reason for this discrepancy is due to differences in population characteristics and healthcare access.

Regarding subgroup analysis, the pooled incidence rate of OIs was higher among adults (9.41 per 100 PYs; 95% CI: 4.88–13.95) compared to children (5.49 per 100 PYs; 95% CI: 4.21–6.77). The observed discrepancy might be explained by differences in healthcare access. For example, children enrolled in HIV care often benefit from early interventions, including programs like prevention of mother‐to‐child transmission (PMTCT) and routine follow‐up services. In contrast, adults living with HIV are more likely to experience delayed diagnosis and late initiation of ART, which may contribute to a higher incidence of OIs.

In this meta‐analysis, adherence to ART medication was found to be a significant predictor of OIs. The pooled analysis showed that HIV patients with poor adherence to ART were 1.42 times more likely to develop OIs compared to those with good adherence (pooled AHR = 1.42; 95% CI: 1.12–1.86). This finding is in line with the studies done in Cameroon [42], Dessie Comprehensive Specialized Hospital [21], Debre markos [40], and Wolaita [8]. Inconsistent treatment enables the virus to replicate, resulting in immune system weakening and a higher susceptibility to infections [12]. Poor adherence to ART significantly increases the risk of OIs, highlighting the importance of adherence support programs. Enhancing patient education, counseling, and follow‐up can improve ART adherence, reduce viral replication, and strengthen immune function, ultimately lowering OI incidence and improving patient outcomes.

Low CD4 count is one of the significantly associated predictors. Hence, in this meta‐analysis, the hazard of occurrence of OIs among < 200 cell/mm CD4 counts was 1.49 times more likely than those with CD4 count greater or equal 200 cell/mm. This finding is in line with the studies done in Dessie Comprehensive Specialized Hospital [21], Amhara region [22], and India [43]. When CD4 counts fall typically below 200 cells/mm^3^, the immune system becomes too weak to fight off pathogens that are normally harmless in healthy individuals [44]. Low CD4 counts (< 200 cells/mm^3^) significantly increase the risk of OIs, emphasizing the critical need for early immune monitoring and timely initiation of ART. Strengthening CD4 count testing and targeted prophylaxis in patients with advanced immunosuppression can reduce OI incidence and improve outcomes.

In this meta‐analysis, the hazard of OIs among bedridden HIV patients increased by 45% as compared with ambulatory HIV patients. This finding is consistent with the study done in Mekelle [7]. Immune suppression impairs the body’s defense mechanisms, reducing its ability to control latent or environmental pathogens. This allows for either reactivation of dormant infections or invasion by new organisms. Unchecked microbial growth leads to tissue damage and systemic spread, culminating in clinical manifestations of OIs [44]. Bedridden HIV patients have a significantly higher risk of OIs, highlighting the need for close clinical monitoring and early intervention in this high‐risk group. Strengthening home‐based care and rehabilitation services can help reduce infection rates and improve patient outcomes in resource‐limited settings.

Advanced WHO clinical stages (Stages 3 and 4) were identified as significant predictors of OIs. This meta‐analysis found that HIV patients in advanced stages had a 57% higher risk of developing OIs compared to those in WHO Stages 1 and 2. The finding is in agreement with the studies done Dessie [21], Wolayita [8], Gondar [45], and South Africa [46]. Advanced HIV severely weakens the immune system by reducing the CD4 cell count. As immune defenses decline, the body becomes vulnerable to OIs [10]. The identification of advanced WHO clinical stages (Stages 3 and 4) as significant predictors of OIs has critical clinical and public health implications. Reducing the proportion of individuals who progress to advanced stages of HIV could significantly lower the incidence of OIs, improve patient outcomes, and support national and global efforts to control the HIV epidemic.

### 4.1. Strength and Limitations

This meta‐analysis offers a valuable and comprehensive estimate of the incidence of OI. The strength of this analysis is that it focuses on the Ethiopian population, providing insights that are tailored to the local context and directly relevant to national health policies. The limitation of this analysis is that certain OIs may be underreported in Ethiopia due to limited diagnostic resources and healthcare infrastructure. This could result in an underestimation of the actual incidence of OIs. Another limitation is publication bias (studies reporting significant or positive results are more likely to be published, whereas those with null or negative findings may remain unpublished). This may lead to an overestimation of the true effect. The third limitation of this SRMA is that the analysis included only studies published in English, which could potentially lead to language bias.

## 5. Conclusion

In this meta‐analysis, the pooled incidence of OIs was 6.96/100 PYs. This finding underscores the significant burden of OIs in the Ethiopian HIV population, highlighting the ongoing challenges in managing HIV‐related complications. Predictors like low CD4 count (< 200 cell/mm^3^), advanced WHO clinical stage, bedridden, and poor adherence were found to be determinants of OIs.

### 5.1. Recommendations

Reducing OIs in PLWH in Ethiopia requires early diagnosis, timely ART initiation, and strong adherence support. Regular CD4 monitoring, targeted care for high‐risk patients, and integrated HIV‐OI services, along with improved health education, are key to improving outcomes.

**Nomenclature**
GRADE: Grading of Recommendations, Assessment, Development, and Evaluation
OIs: Opportunistic infections
PYs: Person‐years
Stata: Statistics and data
WHO: World Health Organization

## Author Contributions

Beyene Zewdu Nigatu: conceptualization, methodology, and writing–review and editing; Beyene Zewdu Nigatu and Amhasilasie Ewunetu Ageze: collection and curation the data, analysis, interpretation, and writing manuscript.

## Funding

No funding was received for this research.

## Ethics Statement

This systematic review and meta‐analysis study does not have direct or indirect involvement of humans or animals. Therefore, ethical approval from IRB was not applicable.

## Consent

The authors have nothing to report.

## Conflicts of Interest

The authors declare no conflicts of interest.

## Supporting Information

Other Supporting Information file

PRISMA _2020_ checklist.

## Supporting information


**Supporting Information** Additional supporting information can be found online in the Supporting Information section.

## Data Availability

All data generated during this study will be made available by the corresponding author upon reasonable request.
